# The C/EBPβ LIP isoform rescues loss of C/EBPβ function in the mouse

**DOI:** 10.1038/s41598-018-26579-y

**Published:** 2018-05-30

**Authors:** Valérie Bégay, Christian Baumeier, Karin Zimmermann, Arnd Heuser, Achim Leutz

**Affiliations:** 1 Tumorigenesis and Cell Differentiation, Max Delbrueck Center for Molecular Medicine, Berlin, 13125 Berlin, Germany; 2 Molecular Physiology of Somatic Sensation, Max Delbrueck Center for Molecular Medicine, Berlin, 13125 Berlin, Germany; 3 Department of experimental Diabetology (DIAB), German Institute of Human Nutrition Potsdam-Rehbruecke (DifE), 14558, Nuthetal, Germany, German Center for Diabetes Research (DZD), München-Neuherberg, Germany; 4 Pathophysiology Group, Max Delbrueck Center for Molecular Medicine, Berlin, 13125 Berlin, Germany; 5 Humboldt-University, Berlin, Institute of Biology, 10115 Berlin, Germany

## Abstract

The transcription factor C/EBPβ regulates hematopoiesis, bone, liver, fat, and skin homeostasis, and female reproduction. C/EBPβ protein expression from its single transcript occurs by alternative in-frame translation initiation at consecutive start sites to generate three isoforms, two long (LAP*, LAP) and one truncated (LIP), with the same C-terminal bZip dimerization domain. The long C/EBPβ isoforms are considered gene activators, whereas the LIP isoform reportedly acts as a dominant-negative repressor. Here, we tested the putative repressor functions of the C/EBPβ LIP isoform in mice by comparing monoallelic WT or LIP knockin mice with *Cebpb* knockout mice, in combination with monoallelic *Cebpa* mice. The C/EBPβ LIP isoform was sufficient to function in coordination with C/EBPα in murine development, adipose tissue and sebocyte differentiation, and female fertility. Thus, the C/EBPβ LIP isoform likely has more physiological functions than its currently known role as a dominant-negative inhibitor, which are more complex than anticipated.

## Introduction

**Figure 1:**
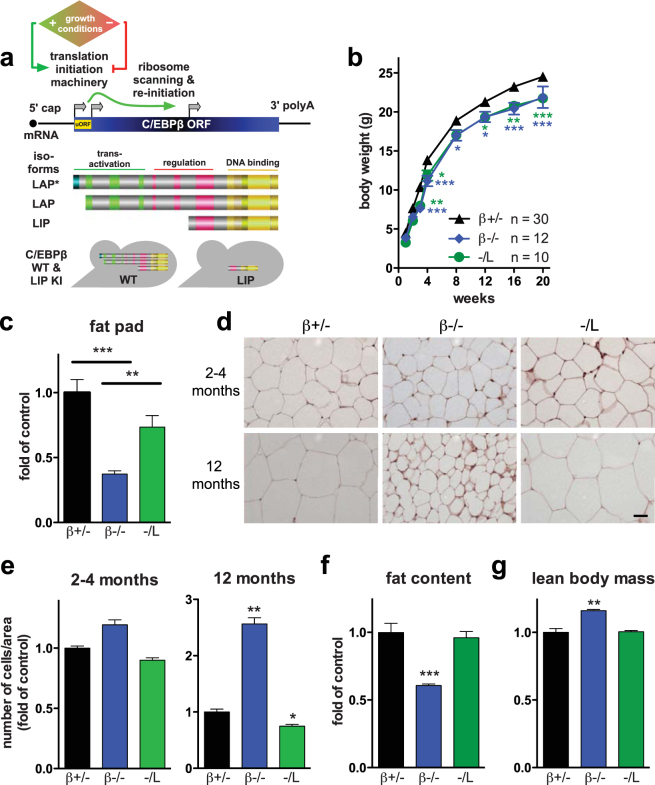
Fat metabolism in *Cebpb* mutant mice. (**a**) Schematic representation of C/EBPβ expression. Environmental conditions regulate the activity of the translation initiation machinery. A cis-regulatory uORF in the single C/EBPβ transcript adjusts initiation of translation at conserved in-frame start sites to generate 3 C/EBPβ isoforms, as indicated. WT mice produce 3 C/EBPβ isoforms, LAP*, LAP, and LIP. The *Cebpb*^*LIP*^ knockin mice produce the truncated C/EBPβ LIP isoform only, which lacks trans-activating and regulatory C/EBPβ regions. (**b**) Body weight analysis of *Cebpb* mutant mice. Body weights were recorded weekly between weeks 1 and 4, and then monthly until sacrifice. Statistical differences were assessed by two-way ANOVA within genotype (P < 0.0001) followed by Bonferroni post-hoc test with each time point. (**c**) Gonadal fat pad weight. (**d**) Histomorphometric analysis of white fat sections of *Cebpb* male mutant mice at 4 and 12 months. Scale bar, 20 μm. (**e**) Quantification of the fat cell area as shown in (**d**). (**f**) Fat content and (**g**) lean body mass. In all panels, bars indicate mean ± SEM. *P < 0.05, **P < 0.01, ***P < 0.001 by Student’s *t*-test. In all panels except where indicated otherwise, mice were 20 weeks of age, n = 6–17 mice per genotype.

The CCAAT enhancer binding protein family of transcription factors (C/EBP) regulates several cellular processes including cell growth, proliferation, differentiation, apoptosis, senescence, and tumorigenesis. C/EBPβ, a member of the C/EBP family, controls cell fate in fat, skin, bone, mammary tubulogenesis, female reproduction, and the innate immune system^1–3^. C/EBPβ is encoded by a single exon gene that is transcribed into a single mRNA and translated by alternative initiation from consecutive in-frame start codons into three protein isoforms, LAP*, LAP, and LIP^4^. The truncated isoform, C/EBPβ LIP, lacks the N-terminal trans-activating and central regulatory regions contained in the long LAP*, LAP isoforms, but may still form dimers via the common C-terminal bZip domain (Fig. 1a). Regulation of the C/EBPβ LAP/LIP ratio plays an important role in liver regeneration, acute phase response, bone homeostasis, metabolic adjustment, monocyte differentiation, tumorigenesis, and mammary gland development^3,5–10^.

The truncated C/EBPβ LIP isoform is widely considered a trans-dominant repressor and a competitive inhibitor that fails to activate transcription, because it lacks an activation domain, and “neutralizes” or “blocks” other C/EBP, ATF, and Jun family members^3,11,12^. Alternative translation of C/EBPβ LIP is required to abrogate C/EBPα-mediated suppression of S-phase entry in liver regeneration^9,13^. C/EBPβ LIP also induces osteoclastogenesis by inhibiting MafB, which acts as an inhibitor of osteoclast differentiation^8^. Further, C/EBPβ LIP is involved in the regulation of cyclin D1 target genes, which are typically repressed by C/EBPβ LAP^14^.

Most of our knowledge on the gene regulatory mechanisms of LIP is derived from tissue culture experiments and transient ectopic expression approaches. However, examination of isoform-specific functions in live animals remains essential for understanding the physiology of C/EBPβ-regulated processes. In mice, deregulated expression of the C/EBPβ LIP isoform causes many diseases related to deregulated cell proliferation, including tumorigenesis^5,15^. Considering the established view that C/EBPβ LIP is a genetic repressor, we recently observed that embryonic fibroblasts (MEF) generated from *Cebpb*^*L/L*^ knockin mice (L/L), expressing only the LIP isoform from its endogenous genetic locus, fail to differentiate into adipocytes in tissue culture (data not shown). Although these data corroborate the conclusions drawn from ectopic expression of C/EBPβ LIP in murine preadipocytic 3T3L1 cells^16^, L/L animals developed white and brown fat. The discrepancy between both systems may be ascribed to the requirement of mitotic clonal expansion of the preadipocytes *in vitro* that is controlled by C/EBPβ LAP*/LAP and inhibited by C/EBPβ LIP^17^, although controversial views do exist^18^. Thus, the physiological roles of C/EBPβ isoforms are difficult to discern from cell culture experiments. We therefore employed a genetic approach to explore the functions of C/EBPβ LIP in live animals.

In this study, we compared the phenotype of monoallelic *Cebpb*^*−/LIP*^ (−/L) mice with that of mice lacking one or both *Cebpb* alleles (*Cebpb*^+/−^, *Cebpb*^*−/−*^). Our results showed that a single *Cebpb LIP* allele rescued fat cell differentiation, prevented hair loss, and partially restored postnatal growth and female fertility. Thus, the physiological functions of truncated C/EBPβ LIP are far more complex than those of an ordinary genetic on/off switch and trans-dominant repressor.

## Results

Tissue culture transactivation assay results suggest that the truncated C/EBPβ isoform LIP exhibits dominant interfering function towards full-length C/EBP proteins. We therefore expected the biological impact of biallelic C/EBPβ LIP expression from its endogenous locus in mice to be more severe than the C/EBPβ knockout phenotype. However, L/L mice did not show hair loss or reduced body weight, two apparent phenotypes of *Cebpb*-deficient mice (Supplementary Fig. 1a,b), at any age. We therefore performed rescue experiments by using mice that expressed only one allele either of WT (*Cebpb*^+/−^, β+/−, used as control), C/EBPβ LIP (*Cebpb*^*−/*L^, −/L), or no C/EBPβ (*Cebpb*^*−/−*^, β−/−), to elucidate the role of the C/EBPβ LIP isoform in live animals.

### *Cebpb*^*−/−*^ mice exhibit lipodystrophy whereas *Cebpb*^*−/LIP*^ mice do not

**Figure 2:**
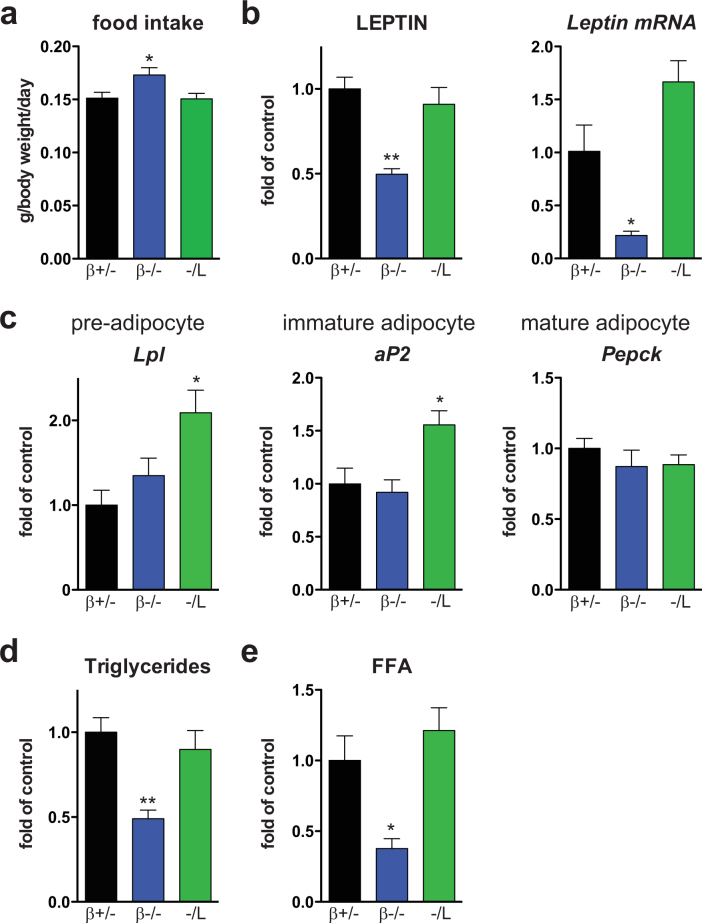
Adipocyte differentiation and lipid homeostasis in *Cebpb* mutant mice. (**a**) Food intake per mouse, normalized to body weight as determined over 4 consecutive days. (**b**) Plasma level of leptin and real-time PCR analysis of leptin in gonadal fat pad. (**c**) Real-time PCR analysis of the adipocyte differentiation markers *Lpl* (pre-adipocytes), *aP2* (immature adipocytes), and *Pepck* (mature adipocytes). (**d**) Plasma levels of triglycerides and free fatty acids (FFA). In all panels, data are presented as mean ± SEM. *P < 0.05, **P < 0.01 by Student’s *t*-test. Mice were 5-month-old (**b**–**d**) or 8-week-old (**a**), n = 4–7 per genotype.

C/EBPβ, together with C/EBPδ, induces the expression of C/EBPα and PPARγ at the onset of fat cell differentiation, and subsequently regulates the expression of adipokines, including leptin^19–25^. Chow-fed age-matched β+/−, −/L, and β−/− mice were generated and monitored over 20 weeks. Western blot analysis revealed that C/EBPβ LAP*, LAP, and LIP isoforms were expressed in white (WAT) and brown (BAT) adipose tissues of β+/− mice. Only the C/EBPβ LIP isoform was found in the WAT and BAT of −/L mice, and none of the isoforms were present in β−/− mice (Supplementary Fig. 2a BAT). The body weight of β−/− and −/L mice was significantly reduced compared to that of β+/− control mice, which were indistinguishable from WT controls (Fig. 1b). Similarly, the body and tibia lengths of β−/− and −/L mice were significantly reduced compared to those of the β+/− controls (Supplementary Fig. 2c,d). *Cebpb*-deficient mice also showed decreased weight of gonadal fat pads and reduced adipocyte cell size compared to β+/− animals (Fig. 1c–e). These phenotypes advanced with age (comparing 2–4 and 12 months; Fig. 1d,e). However, no significant decrease in the weight of gonadal fat pads was found in −/L mice (Fig. 1c), and adipocyte size was indistinguishable at 2–4 months and slightly increased at 12 months compared to β+/− controls (Fig. 1d,e). Similarly, liver, pancreas, and BAT weight of −/L mice were indistinguishable from those of control mice (Supplementary Fig. 2b). Reduced body weight was accompanied by a reduction of total fat mass in β−/− mice but not in −/L mice, as determined by body composition analysis (BCA; Fig. 1f). Furthermore, β−/− mice displayed a significant increase in lean body mass (Fig. 1g), suggesting that the decrease in body weight could be associated with increased energy expenditure, activity, reduced energy intake, or combinations thereof. Further, food intake was increased in β−/− mice (Fig. 2a), consistent with the finding that plasma leptin and leptin transcripts in adipocytes were decreased (Fig. 2b). Unlike in β−/− mice, food uptake and leptin plasma levels in −/L mice were similar to those in the controls (Fig. 2a,b), and a slight increase in leptin mRNA was observed in −/L mice (Fig. 2b).

The decrease in fat cell size in β−/− mice compared with control mice suggests that adipocyte differentiation or function was likely compromised in β−/− animals but not in −/L mutants. Real time PCR analysis showed that lipoprotein lipase (*Lpl*) and the immature adipocyte marker fatty-acid binding protein 4 (*aP2*) expressions were slightly increased in −/L mice, while expression of the mature adipocyte marker phosphoenolpyruvate carboxykinase (*Pepck*) remained unchanged, compared to control littermates, indicating that adipogenic differentiation fully occurred (Fig. 2c). Finally, plasma triglycerides and free fatty acid (FFA) content decreased by at least 50% in β−/− mice but not in −/L mice (Fig. 2d,e, respectively), likely due to the decreased adipose tissue mass in β−/− mice.

The data obtained from β−/− mice are in accordance with previous reports^25,26^. Our data confirm the occurrence of lipodystrophy after removal of all C/EBPβ isoforms. Surprisingly, we now show genetic rescue by a single copy of the truncated C/EBPβ LIP isoform in fat development and homeostasis.

### A single copy of the truncated C/EBPβ LIP isoform rescues alopecia and female fertility of *Cebpb* knockout mice

**Figure 3:**
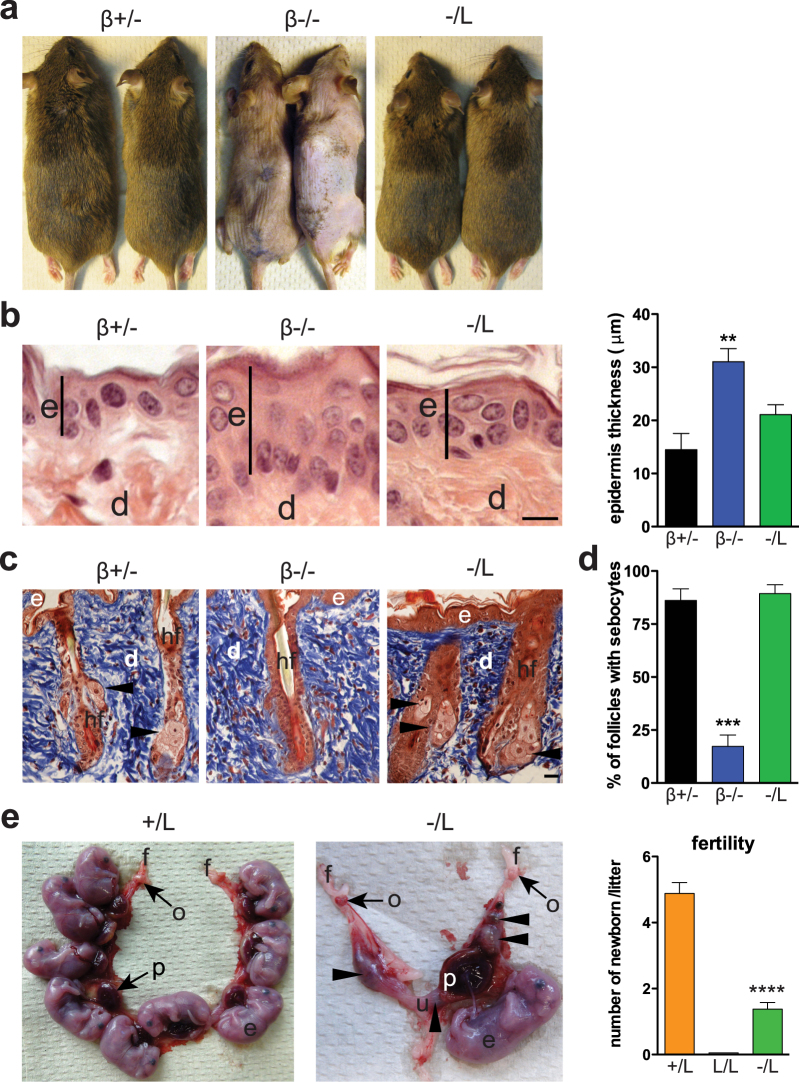
The C/EBPβ LIP isoform rescues keratinocyte and sebocyte differentiation and female fertility. (**a**) Representative photograph of the fur of *Cebpb* male mutant mice. (**b**) H&E staining of dorsal skin showing an increase in epidermal (e) thickness (black line) in β−/− but not in −/L mice. (d) Dermis. Scale bar, 10 μm. Quantification of epidermal thickness is shown on the right, n = 4–7 per group. (**c**) Trichrome staining of dorsal skin showing hair follicle (hf) lacking sebocytes (arrowhead) in  β−/− mice but not in −/L mice. Scale bar, 20 μm. (**d**) Quantification of the number of hair follicle with sebocytes (%) in *Cebpb* mutant mice, n = 8–9 per genotype. (**e**) Representative photographs of the uterus of + /L and −/L females at stage E18.5. Note that in −/L uterus most of the embryos (e) are necrotic (arrowhead) except one, which reached full development. Placenta (p), uterus (u), fat (f), and ovary (o). Quantification of the number of newborn per litter (right panel), n = 5–17 per genotype (also see Supplementary Table 1). In all panels, data are presented as mean ± SEM. **P < 0.01, ***P < 0.001, ****P < 0.0001 by Student’s *t*-test and the mice were 12 months of age in (**a**–**d**).

*Cebpb-*deficient mice showed a progressive hair-fall phenotype and eventually developed hair loss (alopecia) at an advanced age. This phenotype was not observed at any age in −/L mice (Fig. 3a and Supplementary Fig. 1a). Mutations in genes associated with sebaceous gland function lead to loss of hair after several hair cycles^27^. Sebocytes are specialized fat cells of the skin, located in the hair shaft, which produce oily sebum to waterproof and protect hair from drying. C/EBPβ is expressed in sebocytes of the sebaceous glands, where it plays a role in differentiation and lipid metabolism^28^. Germline deletion revealed a role of *Cebpb* in the early epidermal keratinocyte differentiation^29^ and conditional deletion of *Cebpb* in the skin induced a mild phenotype^28,30,31^. We analyzed the skin of age-matched −/L, β−/−, and β+/− mice to determine whether the rescue of white fat cell differentiation extends to sebocytes. *Cebpb-*deficient mice lost their hair (Fig. 3a) and displayed increased epidermal thickness (Fig. 3b). This phenotype was accompanied by reduced number of differentiated sebocytes at 2–4 months of age (Supplementary Fig. 2e), and became more severe with age (Fig. 3c,d). The hair loss phenotype was completely rescued in −/L mice, even at advanced age (Fig. 3a–d). Analysis of the epidermis indicated normalization of epidermal thickness and sebocyte numbers per hair follicle in −/L mice (Fig. 3b,d). Thus, the C/EBPβ LIP isoform is sufficient to sustain both keratinocyte and sebocyte differentiation.

Another severe phenotype observed in female β−/− mice is sterility due to an impairment of granulosa cell differentiation^32^. We observed that L/L female mice were sterile. However, −/L female mice became pregnant but rarely gave birth (Fig. 3e and Supplementary Table 1). Analysis of the uterus of −/L females mice at stage E18.5 showed that several embryos were formed, of which most became necrotic (Fig. 3e). This suggests that the C/EBPβ LIP isoform is sufficient to support fertility to some extent, and indicates dose-dependent functions of LIP.

### A single copy of the truncated C/EBPβ LIP isoform partially rescues the immune phenotype of *Cebpb*^−/−^ mice

C/EBPβ is involved in innate and adaptive immune functions, acute phase response and regulation of cytokine production^7,33–35^. IL6 and TNFα were found elevated in the serum of β−/− mice and splenomegaly was observed at 16 weeks of age^33,34^. Serum levels of IL6 and TNFα are also increased in our young adult β−/− mice (2 month-old), whereas IL6 and TNFα levels remain normal in L/L mice (Supplementary Fig. 3a). At 12 months of age, IL6 and TNFα were significantly increased in both, β−/− and in L/L mice, although variability was observed between animals (Supplementary Fig. 3a). At 5 months of age, β−/− mice showed increased white blood cells (WBC) counts (Supplementary Fig. 3b) and splenomegaly (Supplementary Fig. 3c). The data obtained from β−/− mice are in accordance with previous reports^33,34^. In contrast, one allele of LIP restored WBC (Supplementary Fig. 3b) and normalized or even slightly diminished spleen size (Supplementary Fig. 3c). Moreover, no splenomegaly was observed in +/L or L/L mice at 2 months of age (Supplementary Fig. 1c). Our data suggest that the C/EBPβ LIP isoform to some extent restored immunity that might contribute to the extended lifespan of −/L mice in comparison to β−/− mice (Supplementary Fig. 3d).

### The C/EBPβ LIP isoform functions in conjunction with C/EBPα in fat and skin homeostasis and postnatal survival

**Figure 4:**
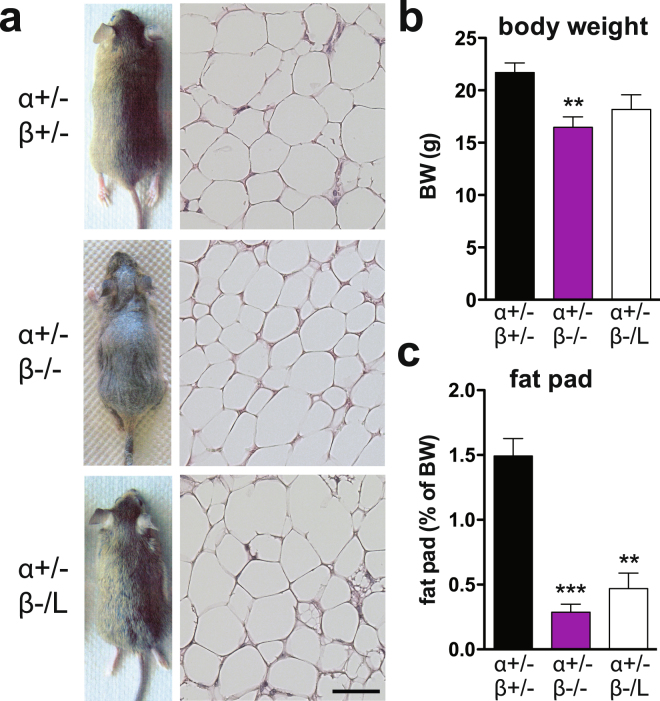
The C/EBPβ LIP isoform functions in tandem with C/EBPα in fat and skin homeostasis. (**a**) Representative photographs of the fur (left panels) of *Cebpa*^+/−^*; Cebpb*^+/−^, *Cebpa*^+/−^*; Cebpb*^−/−^, and *Cebpa*^+/−^*; Cebpb*^*−/L*^ mice. H&E staining of white fat sections of *Cebpb* mutant mice (right panels). Scale bar, 50 μm. (**b**) Body weight and (**c**) fat pad weight of *Cebpa*^+/−^*; Cebpb*^+/−^, *Cebpa*^+/−^*; Cebpb*^−/−^, and *Cebpa*^+/−^*; Cebpb*^*−/L*^ mice at death. In all panels, data are presented as mean ± SEM. **P < 0.01, ***P < 0.001 by Student’s *t*-test. Mice were 8–12 weeks of age, n = 3–7 per genotype.

Of the 6 mammalian C/EBP family members (α, β, δ, ε, γ, and CHOP), C/EBPα and C/EBPβ are essential transcription factors that regulate development and physiology^19,21,30,36^. Previous studies have shown redundancies/specificities of C/EBP family members. For instance, *Cebpb* expressed from the *Cebpa* locus supports postnatal survival and could functionally replace C/EBPα in the liver but not in adipose tissue^37^. Compound loss of *Cebpa* and *Cebpb* in mice (*Cebpa*^−/−^*; Cebpb*^−/−^, or *α*−/−; *β*−/−), caused early embryonic lethality, whereas a single copy of either *Cebpa* in the absence of *Cebpb* or *Cebpb* in the absence of *Cebpa* rescued development, suggesting redundant functions of both C/EBPs during embryogenesis^36^. At the molecular level, C/EBPβ and C/EBPα may form heterodimers that bind to DNA. We therefore determined whether LIP could rescue essential functions of C/EBPβ in conjunction with monoallelic *Cebpa*. We generated and compared mice that expressed a single *Cebpa* allele in the absence of *Cebpb* (*Cebpa*^+/−^*; Cebpb*^−/−^) or in the presence of LIP (*Cebpa*^+/−^*; Cebpb*^*−/*L^ or *Cebpa*^+/−^*; Cebpb*^*L/L*^). The few young adult *Cebpa*^+/−^*; Cebpb*^−/−^ (or *α*+/−; *β*−/−) mice obtained showed a dramatic skin phenotype (Fig. 4a), reduced body weight (Fig. 4b), reduced fat pad weight (Fig. 4c) with fat pad lipoatrophy (Fig. 4a), increased liver weight (Supplementary Fig. 4a), and splenomegaly (Supplementary Fig. 4b), and had to be euthanized within 2–3 months because of severe cachexia, infection, and skin lesions. In contrast, mice with a single copy of *Cebpa* and a single copy of *Cebpb* LIP (*Cebpa*^+/−^*; Cebpb*^*−/L*^ or *α*+/−; −/L) showed almost complete rescue of the hair phenotype (Fig. 4a). Interestingly, *Cebpa*^+/−^*; Cebpb*^*−/L*^ mice showed a substantial decrease in body weight (Fig. 4b), while liver, BAT and spleen weight remained unaltered in comparison to compound heterozygote *Cebpa*^+/−^*; Cebpb*^+/−^ mice (Supplementary Fig. 4a,b). Fat pad morphology showed adipocytes of various sizes that resembled the adipocyte morphology of control littermates (Fig. 4a), but the overall amount of fat pad was reduced (Fig. 4c). Importantly, a single copy of *Cebpa* in *Cebpb*^−/−^ mice only marginally supported birth and postnatal survival (mendelian ratio of around 1.2% instead of 12.5% at birth; Supplementary Table 2 and^36^). Interestingly, mice expressing one *Cebpa* allele in *Cebpb*^*L/L*^ were born at only slightly reduced mendelian ratio (9.09% instead of 12.5%, Supplementary Table 2), displayed no macroscopic alterations, and survived at least 6–12 months (data not shown). Mice expressing one *Cebpa* allele in *Cebpb*^*−/L*^ were born at a lower mendelian ratio (5.43% instead of 12.5%, Supplementary Table 2), suggesting dose-dependent effects of the C/EBPβ LIP isoform in postnatal survival. Taken together, our results indicated that a single *Cebpb* LIP isoform allele was sufficient to rescue survival and hair loss. Thus, the LIP isoform likely acts in conjunction with monoallelic C/EBPα to rescue several postnatal functions in mice.

## Discussion

The truncated LIP isoform of C/EBPβ lacks a transactivation domain, and has been classified as a repressor of transcription and an antagonist of the long C/EBP isoforms.

In this study, we compared the phenotype of monoallelic *Cebpb*^*−/LIP*^ mice with that of mice lacking one or both *Cebpb* alleles. Our results showed that several defects caused by the absence of the *Cebpb* gene could be compensated by the truncated C/EBPβ LIP isoform. The functions of C/EBPβ LIP observed in live animals are difficult to be explained by mechanisms of trans-dominant interference. Thus, the classification of C/EBPβ LIP as a dominantly interfering transcription factor likely depends on temporal and cellular context and requires to be revisited in light of the physiological data presented in this report.

Correct temporal expression of the C/EBP family members C/EBPβ, C/EBPδ, and C/EBPα, is crucial for the execution of the adipogenic differentiation program^19–21,23^. Reduced fat pad mass was observed in *Cebpb*^−/−^*; Cebpd*^−/−^ mice^25,26^, and inhibition of DNA binding of all C/EBP members in fat tissue led to a biphasic lipodystrophy syndrome with loss of WAT at birth^38^. These results were largely in agreement with the expected role of C/EBPs in murine adipogenesis^39^. CCAAT/enhancer binding protein homologous protein (CHOP) has been identified as a negative regulator of C/EBP function and CHOP-deficient mice show increased body weight and adiposity compared to littermate controls^39,40^. Further, in tissue culture, CHOP could inhibit C/EBPα and C/EBPβ function during fat cell differentiation^41,42^. Therefore −/L and L/L mice could be expected to show similar fat phenotypes. However, our data show that the C/EBPβ LIP isoform is sufficient to sustain fat and skin development and homeostasis along with C/EBPα. According to trans-dominant functions of C/EBPβ LIP deduced from ectopic expression experiments, −/L mice would have been expected to show a more dramatic phenotype on fat tissue, skin and even acceleration of early postnatal death due to hypoglycemia as in *Cebpa*^−/−^ mice^30,43^. Moreover, a more severe immune phenotype, aggravating effects of highly deficient myelopoiesis was expected, as seen in compound C/EBPβ and C/EBPε knockouts^44^. However, with the stipulation that animals were kept under specific-pathogen-conditions, −/L mice did not show gross immune defects and survived to at least 20 months, much longer than *Cebpb*^−/−^ mice. Finally, *Cebpa*^+/−^*; Cebpb*^*−/L*^ mice clearly showed rescue of pathology and lethal phenotypes, as compared to *Cebpa*^+/−^*; Cebpb*^−/−^ mice, such as restoration of the skin phenotype. It is tempting to speculate that complementation of C/EBPβ deficiency by LIP isoform occurs through partially restored immune functions, however, C/EBPβ LIP failed to rescue many innate and adaptive immune defects^5,7,45^. Altogether, our data suggest residual essential functions of the truncated C/EBPβ LIP isoform. Surprisingly, expression of C/EBP target genes in adipose tissues (WAT and BAT) of −/L mice were similar to those of *Cebpb*^*−/*ΔuORF^ mice, expressing only LAP*/LAP (Supplementary Fig. 5).

C/EBPβ binds directly to the leptin promoter and activates leptin expression^23,25^, however, our data suggest that the C/EBPβ LIP isoform is sufficient to fulfill leptin gene regulation, since both leptin transcript expression in WAT and leptin levels in the blood in −/L mice remained normal. Architectural and/or functional features of the conserved region 7 (CR7), DNA binding bZip domain, and residues in the C-terminus common to all C/EBPβ isoforms (Fig. 1a) are thus sufficient for functional complementation. Phosphorylation by MAPK/ERK1,2 of C/EBPβ at threonine 188 and in the leucine zipper are important for DNA binding and activation of the adipogenic program and these sites also reside in C/EBPβ LIP isoform^20,46,47^. Furthermore, both, β−/− and L/L female mice failed to become pregnant (Supplementary Table 1 and^32^), however, *Cebpb*^ΔuORF/ΔuORF^ female mice expressing only LAP*/LAP could reproduce with the normal mendelian ratio^9^. Fertility has been linked to MAPK/ERK1,2-dependent phosphorylation of C/EBPβ to activate critical genes in the oocyte^48^. Interestingly, we observed that −/L female mice could become pregnant but rarely carried to full term. These data suggest that a single LIP allele (in *Cebpb*^*−/*L^) could also support fecundity but not late pregnancy. The fact that −/L but not L/L female mice could become pregnant emphasizes the importance of LIP isoform dosage *in vivo* and potentially its balanced co-function with C/EBPα. The dosage of the C/EBPβ LIP isoform expression thus also emerges as an important quantitative aspect to be addressed, shifting the focus to adjustment of isoform ratios by the C/EBPβ uORF. How the level of LIP expression influence target gene expression still remains to be determined. Taking advantage of the mono versus bi-allelic +/L and L/L mice no antagonal effects between one or two copies of LIP were found in preliminary studies on potential C/EBPβ target gene expression in B cells (Supplementary Fig. 6), however, such analysis will have to be extended to the whole animal physiology. Furthermore, the MAPK/ERK1,2 sites that are retained in C/EBPβ LIP could further be studied in the future to help explain the rescue of *Cebpb*^*−/*L^ female fertility and other phenotypes.

Taken together, our and published data warrant reevaluation of the functions of the truncated isoforms of both, C/EBPβ (this publication) and C/EBPα^49^. Future work will have to address how C/EBPβ LIP or the corresponding truncated C/EBPα p30 isoform might compensate for loss of the entire genes. Of particular future attention are the genomic distribution of C/EBP isoforms, their interactome and genomic co-localization with co-activating or repressing machinery, in addition to large scale structural alterations of chromatin.

In summary, data presented here strongly suggest that the C/EBPβ LIP isoform that lacks the transactivation domain is more complex than a trans-dominant inhibitor and rescues essential functions of native C/EBPβ by mechanisms that remain to be explored. A full understanding of the complexity of C/EBPα and C/EBPβ regulation at the physiological-mechanistic level clearly remains challenging but nevertheless will be important to eventually develop novel therapeutic strategies for the successful treatment of C/EBP-related disorders in homeostasis and tumorigenesis.

## Material and Methods

### Animals

*The Cebpb and Cebpa* knockout strains and *Cebpb*^*LIP*^ knockin mice have been previously described^8,32,43^. Mouse strains were maintained on a 129 × C57BL/6N genetic background, because *Cebpb*^*L/L*^ and *Cebpb*^−/−^ mice died in the C57BL/6N genetic background. For more details about mouse strains, see Supplementary Methods in SI. Data presented were obtained from female mice, unless indicated otherwise. Mice were fed ad libitum with standard diet and water on a 12-h light-dark cycle. Animals were housed in a pathogen-free facility at the MDC, Berlin. All procedures and animal experiments were conducted in compliance with protocols approved by the institutional Animal Care and Use Committee Landesamt für Gesundheit und Soziales Berlin (LAGeSo). Mice were sacrificed by cervical dislocation. All efforts were made to minimize animal suffering.

### Histology

Fat pads were collected, fixed overnight in 4% paraformaldehyde, embedded in paraffin, sectioned at 4 μm, and stained with hematoxylin & eosin according to the standard protocol. Images were acquired using a Zeiss AxioCam Hr camera. Five randomly chosen microscope fields were photographed at 200X magnification for each animal, and analyzed using the Zeiss AxioVision Software program (version 4.2).

### Biochemical assay

Blood was collected by heart puncture of non-fasted mice after killing by cervical dislocation. Serum leptin was measured using the mouse leptin RIA kit (Millipore). Serum triglycerides (LabAssayTM Triglyceride, Wako-chemicals) and free fatty acid (NEFA-HR(2), Wako-chemicals) were determined using colorimetric assays.

### Body composition

Body composition was measured in awaken 20-week-old female mice by using Time Domain Nuclear Magnetic Resonance (Minispec LF90 II - Bruker BioSpin). Testing was conducted in randomly fed mice that were fasted for 3 h before measurement.

### Food intake

Food intake was measured in female mice at 8 weeks. Mice were caged individually and adapted for at least 48 h before measuring food intake. Food consumption was determined for 4 consecutive days, expressed as g/day, and normalized to the body weight of the mice.

### RNA isolation and real-time quantitative PCR analysis

Total RNA was isolated using TriPure isolation reagent (Roche). Total RNA (2 μg) was treated with DNAse (Invitrogen) and reverse transcribed at 42 °C by using Superscript II (Invitrogen). Real-time quantitative PCR was performed on a LightCycler type II (Roche) using the SYBR Green Master Mix (Roche). Expression of 36B4 or Tbp was used to normalize individual RNA expression levels. The data were expressed as relative RNA expression levels, calculated using the comparative CT method. The control expression level was set at 1. Sequences of primers pairs used can be provided upon request.

### Cell culture and immunoblotting

Mouse embryonic fibroblasts and 3T3-L1 murine preadipocytes were cultured in DMEM supplemented with 10% FBS (Invitrogen). Tissues and cells were lysed with 8 M urea and proteins were analyzed by SDS/PAGE/protein blotting using rabbit C/EBPβ antibody (C19, Santa Cruz) and mouse anti-β-tubulin (2-28-33, Sigma), horseradish peroxidase-conjugated secondary antibodies, and chemiluminescence detection (Amersham Biosciences).

### Statistical analysis

All data are expressed as mean ± SEM. Data were first tested for normal distribution. Statistically significant differences between groups were determined using the unpaired two-tailed Mann-Whitney’s test unless indicated otherwise (Prism 5, GraphPad Software). P-value < 0.05 was considered to be statistically significant.

## Electronic supplementary material

Supplementary information
