# Supplementary material for: The C/EBPβ LIP isoform rescues loss of C/EBPβ function in the mouse
Source: Sci Rep. 2018 May 30;8:8417. doi: 10.1038/s41598-018-26579-y (PMC5976626; doi:10.1038/s41598-018-26579-y)
Supplement: Supplementary file 1 — Supplementary information [file 41598_2018_26579_MOESM1_ESM.pdf]

Supplementary Information

The C/EBPβ LIP isoform rescues loss of C/EBPβ function in the mouse

Valérie Bégay[1,2*], Christian Baumeier[1,3], Karin Zimmermann[1], Arnd Heuser[4] and Achim Leutz[1,5*]

Supplementary methods

Generation of *Cebpb* mutant mice

Cebpb$^{L/L}$ knockin mice expressing only LIP isoform have been previously described (Ref 8: Smink et al., 2009). Briefly, nucleotides 91 to 550 encoding LAP* and LAP N termini were removed from the endogenous genetic locus. Thus, LAP* and LAP isoforms can not be generated.

Cebpb$^{-/-}$ knockout mice were generated by replacing the *Cebpb* single exon by the neomycin cassette (Ref 32: Sterneck et al., 1997).

Cebpb$^{\Delta uORF/\Delta uORF}$ knockin mice expressing only LAP*/LAP isoforms have been previously described (Ref 9: Wethmar et al., 2010). Briefly, the *Cebpb* uORF initiation site was mutated in the endogenous genetic locus leading to no expression of LIP isoform in this mutant mice.

Cebpb$^{-/L}$ knockin mice were generated by crossing *Cebpb*$^{-/-}$ knockout male mice with *Cebpb*$^{+/L}$ female mice.

Cebpb$^{-/\Delta uORF}$ knockin mice were generated by crossing *Cebpb*$^{-/-}$ knockout male mice with *Cebpb*$^{+/\Delta uORF}$ female mice.

Histology and quantification of skin parameters

Dorsal skin samples were collected, fixed overnight in 4% paraformaldehyde, embedded in paraffin, sectioned at 4-8 μm, and stained with hematoxylin & eosin or Trichrome (Masson) staining kit (Sigma) according to the manufacturer protocol. Images were acquired using a Zeiss AxioCam Hr camera. For quantification of the epidermis, five randomly chosen microscope fields were photographed at 400X magnification for each animal, and analyzed using the Zeiss AxioVision Software program (version 4.2). For the quantification of the number of follicle with sebocytes, five randomly chosen microscope fields were photographed at 200X magnification for each animal.

Blood count analysis

After sacrifice of the animals, blood samples were quickly obtained from the heart and collected into EDTA coated tubes. The blood cell counts were measured with an automated veterinary hematological counter Scil Vet abc (SCIL GmbH, Viernheim, Germany) with software optimized for mouse blood parameters.

Biochemical assay

Blood samples was collected by heart puncture and serum samples were obtained after centrifugation at 2,000g for 10 minutes at 4°C. Serum IL6 and TNFα protein levels were determined using a Mouse IL6 and TNFα ELISA kits (BD Biosciences) according to the manufacturer's instructions.

B cells isolation and real-time quantitative PCR analysis

Isolation of B cells from +/L and L/L mice was described previously (Ref 5: Bégay et al., 2015). RNA preparation and real time quantitative PCR analysis were performed as described in the main text. Expression of *Gapdh* was used to normalize individual RNA expression levels. The data were expressed as relative RNA expression levels, calculated using the comparative CT method. The control expression level was set at 1. Sequences of primers pairs used can be provided upon request.

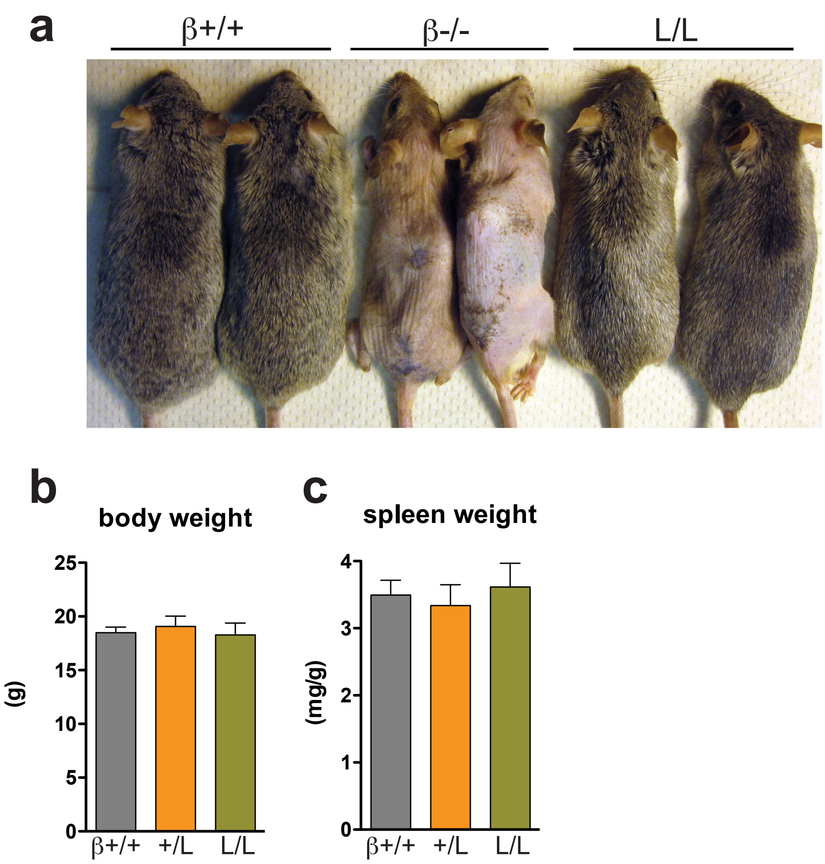

Supplementary Figure 1: Phenotypes of *Cebpb*^{LIP} knockin mice.
(**a**) Representative photograph showing the fur of L/L male mice in comparison to β-/- and β+/+ male mice at 12 months of age. The same β-/- mice are shown in Figure 3A. (**b**) Body weight (in g) and (**c**) weight of spleen (plotted as spleen weight / body weight, mg/g) of *Cebpb*^{LIP} knockin mice at 8 weeks of age, Body weight: n = 7-10 per genotype; spleen: n = 6-12 per genotype. Bars indicate mean ± SEM.

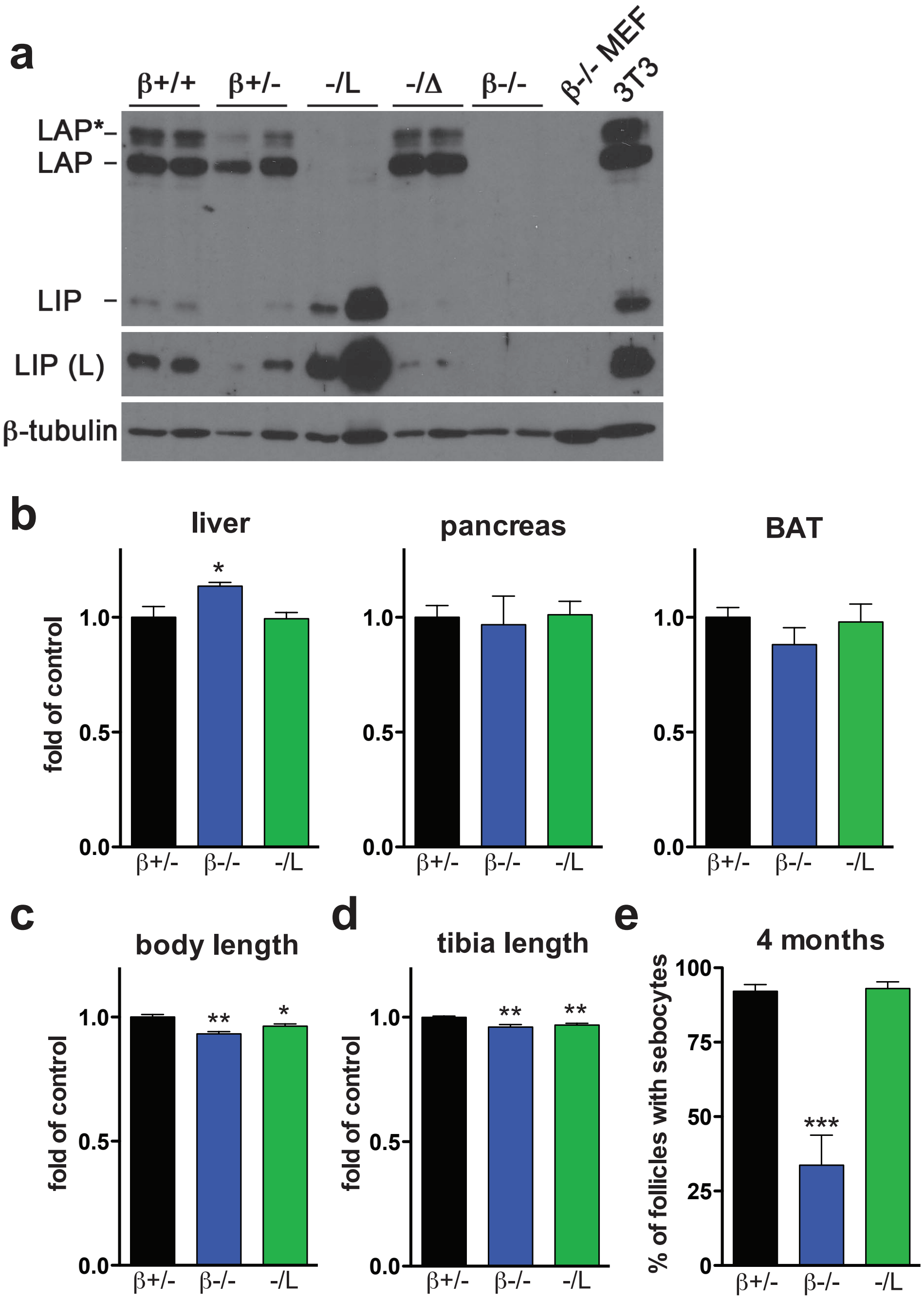

Supplementary Figure 2: Ancillary phenotypic analysis of *Cebpb* mutant mice.
(**a**) Brown adipose tissue (BAT) isolated from 4-month-old *Cebpb* mutant mice were lysed in 8 M urea lysis buffer and analyzed by western blotting for expression of the C/EBPβ isoforms LAP*, LAP, and LIP (as indicated). 3T3-L1 cells and β-/- MEF were used as positive and negative controls, respectively. β-tubulin was used as internal loading control. *Cebpb*$^{-/\Delta uORF}$ mice (-/Δ) only express the long isoforms LAP* / LAP and served as positive control for the expression of LAP* / LAP isoforms. *Cebpb* mutant mice used: -/L, β-/-, β+/-, -/Δ, and β+/+ mice. LIP(L) panel shows LIP isoform expression detected after longer exposure time. Uncropped photographs of the protein blots are shown in Supplementary Figure 7. (**b**) Weight of liver, pancreas, and BAT, (**c**) body length and (**d**) tibia length of *Cebpb* mutant mice at 20 weeks as indicated, n= 7-17 per genotype. (**e**) Quantification of the number of hair follicles with sebocytes (%) at 2-4 months in *Cebpb* mutant mice, n = 7-11 per genotype. Data are presented as mean ± SEM. *P < 0.05,**P < 0.01, ***P < 0.001 by appropriate Student's *t*-test.

Supplementary Table 1: Postnatal survival and lethality of newborns from matings of *Cebpb*^{LIP} female mice.

Mating scheme			N° of animals	N° of animals	Genotypes[a] and Mendelian ratios (%)			
male	female	N° of litters	born	survived				
With heterozygous LIP females								
					**+/+** (25)	$\beta^{+/L}$ (50)	$\beta^{L/L}$ (25)	
$\beta^{+/L}$	$\beta^{+/L}$	48	ND	324	26.2	56.2	17.6	
					**+/+** (25)	$\beta^{+/-}$ (25)	$\beta^{-/L}$ (25)	$\beta^{+/L}$ (25)
$\beta^{+/-}$	$\beta^{+/L}$	5	ND	33	27.27	33.33	15.15	24.24
						$\beta^{+/-}$ (50)	$\beta^{-/L}$ (50)	
$\beta^{-/-}$	$\beta^{+/L}$	52	ND	325		57.23	42.77	
						$\beta^{+/L}$ (50)	$\beta^{L/L}$ (50)	
$\beta^{L/L}$	$\beta^{+/L}$	57	ND	299		64,21	35,79	
With homozygous LIP females								
						$\beta^{+/L}$ (50)	$\beta^{L/L}$ (50)	
$\beta^{+/L}$	$\beta^{L/L}$	0	0[b]	0		0	0	
With rescued LIP females								
						$\beta^{+/-}$ (50)	$\beta^{-/L}$ (50)	
$\beta^{+/-}$	$\beta^{-/L}$	16	22	0		0	0	

[a]The different genotypes obtained from intercrosses of *Cebpb* mice are indicated in the column headings. The genotypes are determined at weaning. The expected Mendelian ratios are given in parentheses in the column heading. [b]Mating was confirmed by the presence of vaginal plugs, but the females did not appear to become pregnant and no pups were delivered thereafter. Five L/L females were tested.

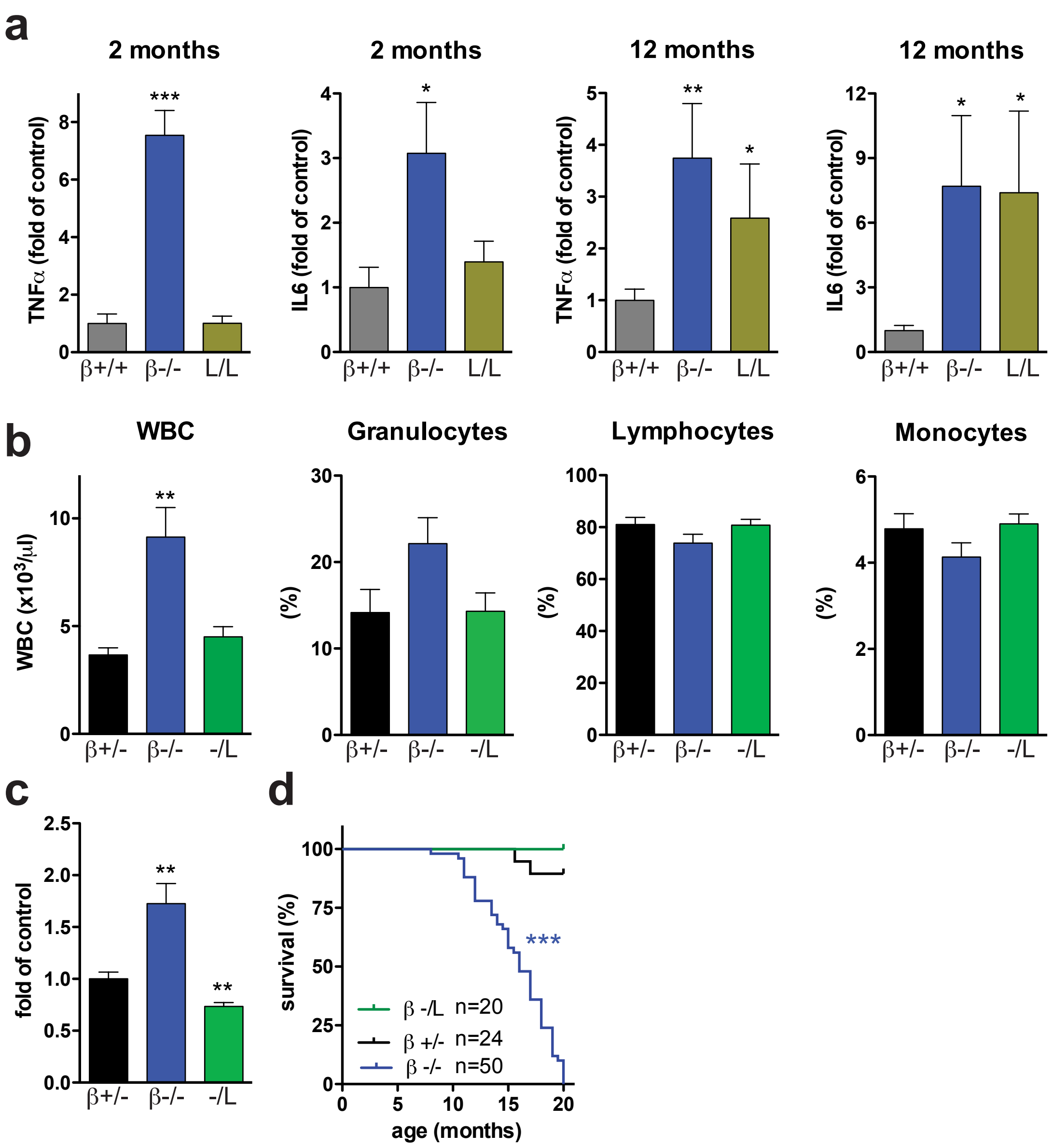

Supplementary Figure 3: Determination of cytokines and immune cells in mice with various *Cebpb* genotypes. (**a**) Serum TNFα and IL6 levels at 2 months and at 12 months in *Cebpb* mutants as indicated. n= 6 to 14 per genotype. (**b**) Blood cell counts in the *Cebpb* mutant mice as indicated at 5 months. WBC: white blood cells. n= 5 to 8 per genotype. (**c**) Weight of spleen of *Cebpb* mutants at 5 months. n= 7 to 9 per genotype. (**d**) Survival curves of *Cebpb* mutants until 20 months of age. Log-rank test ***P< 0.001. Cohort of mice: β +/- n= 24; β -/-, n= 50; -/L, n= 20. (**a-c**) Data are presented as mean ± SEM. *P< 0.05, **P< 0.01,***P< 0.0001 by Student's *t*-test.

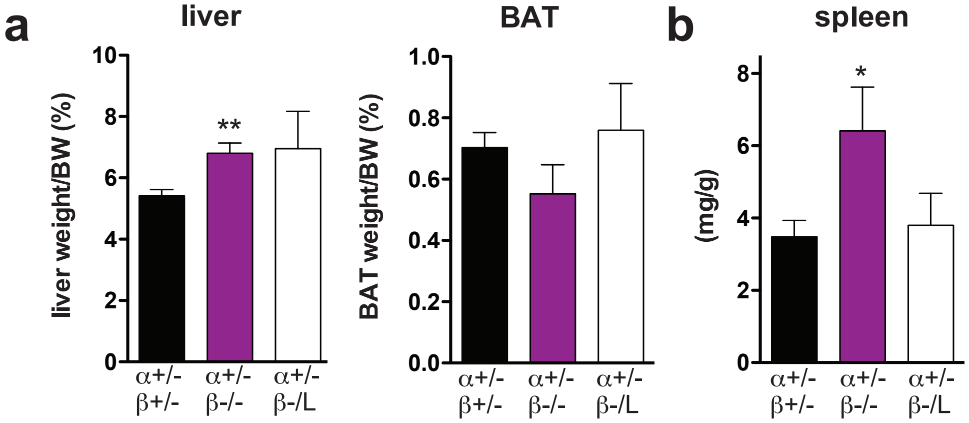

Supplementary Figure 4: Weight of tissues of compound *Cebpab* mutant mice. (**a**) Weight of liver and BAT (plotted as tissue weight (g)/body weight (g) X100) and (**b**) weight of spleen (plotted as spleen weight in mg/ body weight in g) in *Cebpa*$^{+/-}$; *Cebpb*$^{+/-}$ (or α+/-; β+/-), *Cebpa*$^{+/-}$; *Cebpb*$^{-/-}$ (or α+/-; β-/-) and *Cebpa*$^{+/-}$; *Cebpb*$^{-/L}$ (or α+/-; -/L) mice as indicated. n = 3-7 per genotype. Data are presented as mean ± SEM. *P < 0.05, **P < 0.01 by Student's *t*-test.

Supplementary Table 2: postnatal survival and lethality of compound *Cebpa* and *Cebpb* mutant mice.

Mating scheme	N° of animals	Genotypes and Mendelian ratios (%)								
		**+/+**	$\alpha^{+/-}$	$\alpha^{+/-}$ $\beta^{+/L}$	$\alpha^{+/-}$ $\beta^{L/L}$	$\alpha^{-/-}$	$\alpha^{-/-}$ $\beta^{+/L}$	$\alpha^{-/-}$ $\beta^{L/L}$	$\beta^{+/L}$	$\beta^{L/L}$
$\alpha^{+/-}$; $\beta^{+/L}$ X		(6.25)	(12.5)	(25)	(12.5)	(6.25)	(12.5)	(6.25)	(12.5)	(6.25)
$\alpha^{+/-}$; $\beta^{+/L}$	77	7.79	19.48	44.16	9.09	0	0	0	16.88	2.6
			$\alpha^{+/-}$ $\beta^{+/-}$	$\alpha^{+/-}$ $\beta^{+/L}$	$\alpha^{+/-}$ $\beta^{-/L}$	$\alpha^{+/-}$ $\beta^{-/-}$	$\beta^{-/L}$	$\beta^{-/-}$	$\beta^{+/L}$	$\beta^{+/-}$
$\alpha^{+/-}$; $\beta^{+/-}$ X			(12.5)	(12.5)	(12.5)	(12.5)	(12.5)	(12.5)	(12.5)	(12.5)
$\alpha^{+/+}$; $\beta^{-/L}$	92		14.13	21.74	5.43	1.09	16.3	14.13	11.96	15.22
		**+/+**	$\alpha^{+/-}$ $\beta^{+/-}$	$\alpha^{-/-}$ $\beta^{-/-}$	$\alpha^{+/-}$ $\beta^{-/-}$	$\alpha^{-/-}$	$\alpha^{-/-}$ $\beta^{+/-}$	$\beta^{-/-}$	$\alpha^{+/-}$	$\beta^{+/-}$
$\alpha^{+/-}$; $\beta^{+/-}$ X		(6.25)	(25)	(6.25)	(12.5)	(6.25)	(12.5)	(6.25)	(12.5)	(12.5)
$\alpha^{+/-}$; $\beta^{+/-}$	[b]247	8.9	44.5	0	1.21	0	0	7.29	15.4	21.9

[a] Genotypes obtained from intercrosses of *Cebpa* and *Cebpb* mutant mice are indicated in the column headings. The genotypes are determined at weaning except [b] at birth. The expected Mendelian ratios are shown in parentheses.

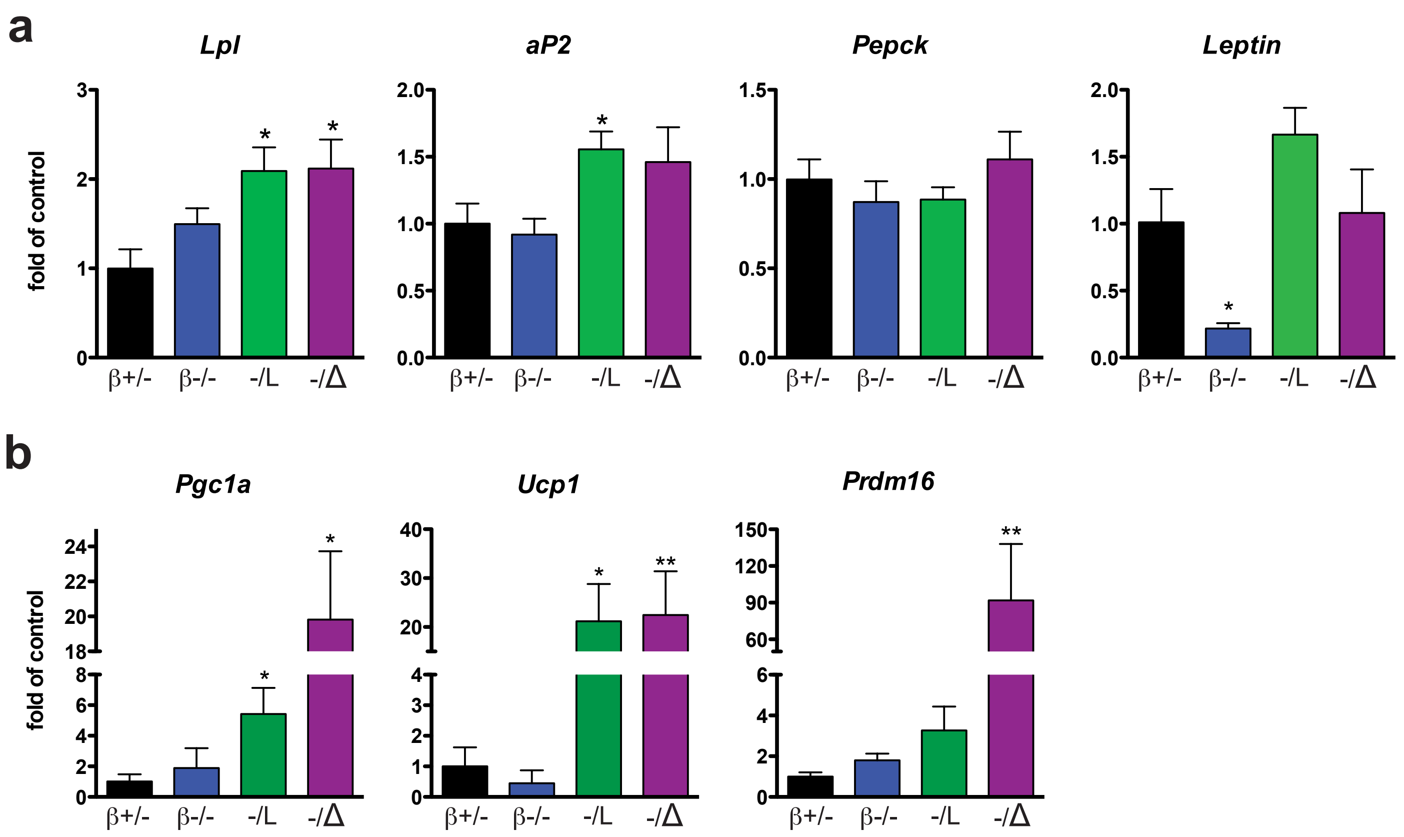

Supplementary Figure 5. RT-PCR analysis of C/EBP target genes in adipose tissue of *Cebpb* mutant mice. Relative mRNA expression of C/EBP target genes in WAT (**a**) and BAT (**b**) similarly increased in -/L and *Cebpb*$^{-/\Delta uORF}$ mice (-/Δ), the latter only express the long isoforms LAP* / LAP. (**a**) n = 3 to 7 per genotype. (**b**) n = 3 to 6 per genotype. Data are presented as mean ± SEM. *P< 0.05, **P< 0.01 by Student's *t*-test. Note that data obtained on β+/-, β-/- and -/L mice showed in (**a**) are also shown in Figure 2b, c.

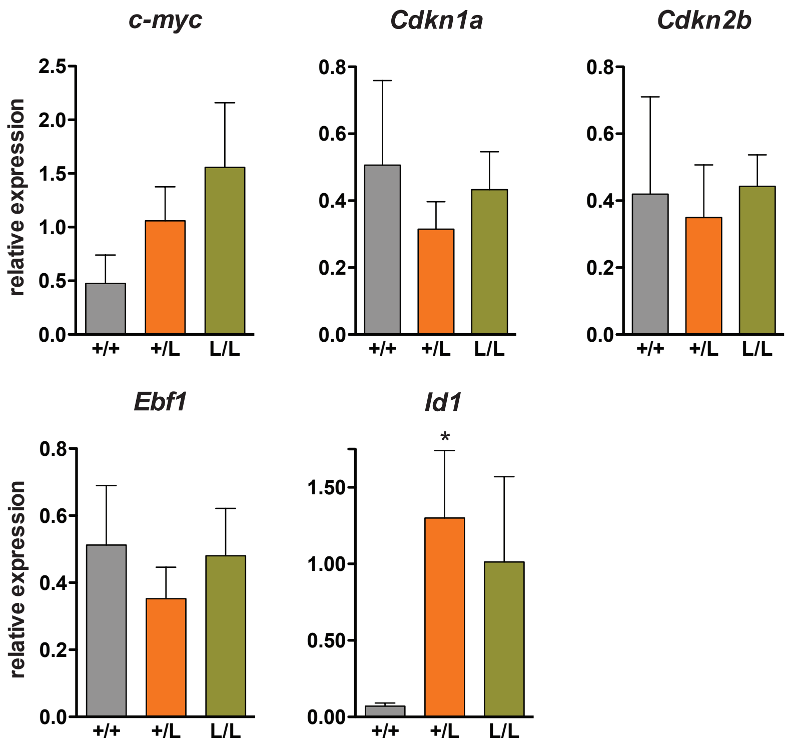

Supplementary Figure 6: RT-PCR analysis of potential C/EBPβ target genes in B cells of +/L and L/L mutant mice at 12 months of age. n= 3 to 4 per genotype. Data are presented as mean ± SEM. *P< 0.05 by Student's *t*-test.

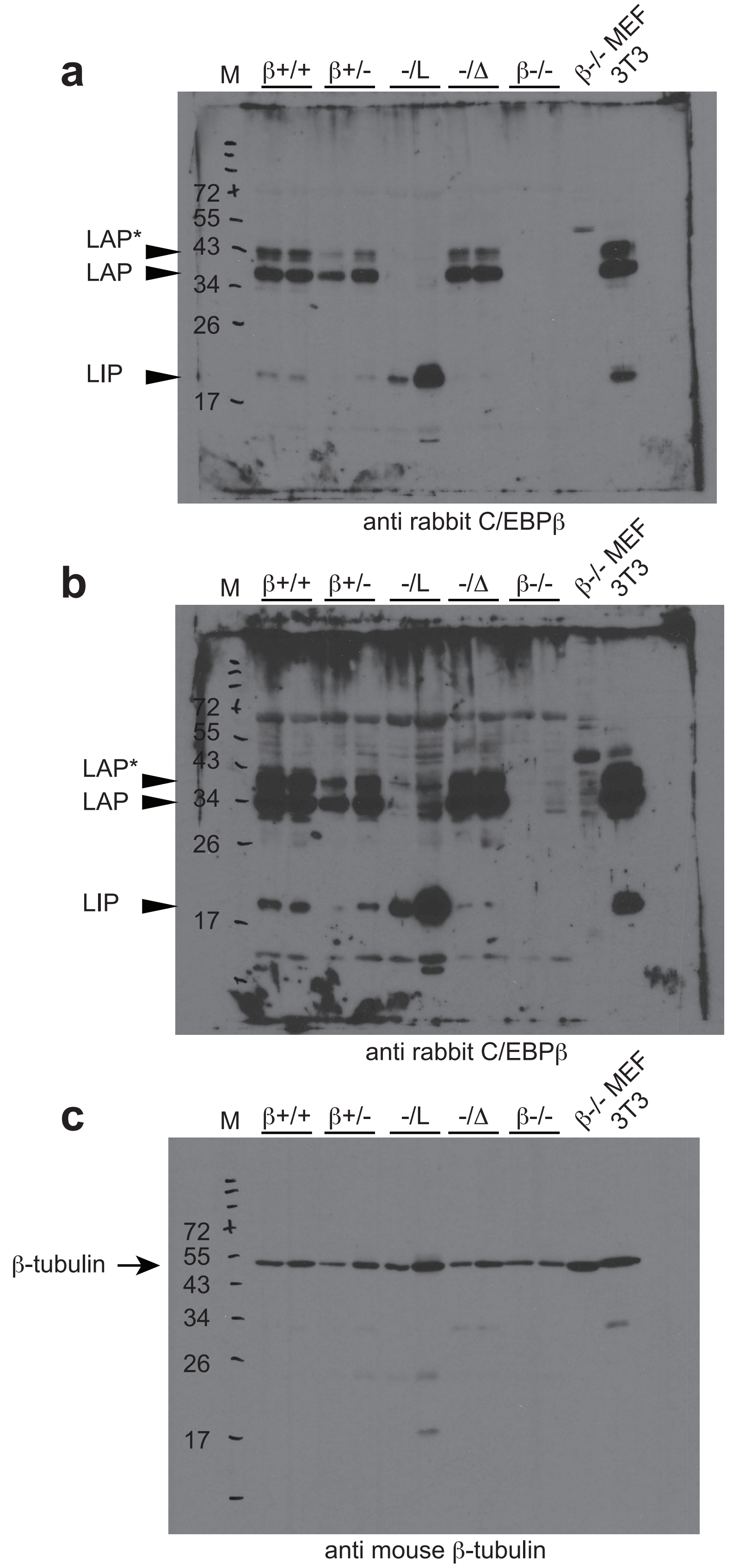

Supplementary Figure 7: Uncropped photographs of the protein blots shown in Supplementary Figure 2a. Immunodetection of C/EBPβ antibody (C19) was revealed by ECL chemiluminescence (GE healthcare) with short exposure (**a**) or with long exposure time for improvement of the detection of the LIP isoform (**b**). (**c**) Immunodetection of β-tubulin after stripping of the PVDF membrane.
